# The Thromboembolic Continuum in Transcatheter Mitral Valve Repair: A Comprehensive Review

**DOI:** 10.3390/jcm15093227

**Published:** 2026-04-23

**Authors:** Nikolaos Manganiaris, Kyriakos Dimitriadis, Kyriaki Mavromoustakou, Nikolaos Pyrpyris, Eleni Adamopoulou, Daphne Pitsiori, Eirini Beneki, Panagiotis Iliakis, Eirini Dris, Polykarpos Christos Patsalis, Konstantinos Aznaouridis, Konstantinos Tsioufis

**Affiliations:** 1 First Department of Cardiology, School of Medicine, National and Kapodistrian University of Athens, Hippokration General Hospital, 115 27 Athens, Greece; manganiaris.n2003@gmail.com (N.M.); mavromoustakoukiriaki@yahoo.gr (K.M.); npyrpyris@gmail.com (N.P.); 99elenaadam@gmail.com (E.A.); daphne.pitsiori@yahoo.com (D.P.); e.beneki@hotmail.com (E.B.); panayiotisiliakis@gmail.com (P.I.); iredris@hotmail.com (E.D.); conazna@yahoo.com (K.A.); ktsioufis@gmail.com (K.T.); 2 Department of Cardiology, Lausanne University Hospital, 1005 Lausanne, Switzerland; 3 Department of Medicine, Division of Cardiology, Angiology and Internal Emergency Medicine, Ruhr University Bochum, Knappschaft Kliniken University Hospital Bochum, 44892 Bochum, Germany; polykarpos.patsalis@ruhr-uni-bochum.de

**Keywords:** mitral regurgitation, transcatheter edge-to-edge repair, stroke, atrial cardiomyopathy, subclinical ischemia, anticoagulation

## Abstract

Mitral transcatheter edge-to-edge repair (M-TEER) has emerged as a cornerstone in the management of severe mitral regurgitation, serving as a robust, low-risk alternative to conventional mitral valve surgery. Although thromboembolic risk remains a critical clinical challenge, that varies significantly across the clinical continuum, from pre-procedural substrates to post-procedural management. This review highlights the role of atrial cardiomyopathy in creating a prothrombotic milieu even prior to intervention, while during the procedure, device time emerges as a potentially dominant independent predictor of embolic burden, marking the periprocedural window as the period of peak hazard. Furthermore, this article addresses the notable disparity between the near-universal presence of subclinical ischemic lesions on magnetic resonance imaging and the infrequent incidence of overt neurological deficits. As the post-procedural phase is considered, we discuss the shift from standardized antithrombotic protocols to individualized strategies and the potential role of concomitant left atrial appendage occlusion. Ultimately, integrating these stage-specific clinical and procedural determinants with emerging technologies—like digital twins and artificial intelligence—represents a promising frontier for mitigating embolic risks, optimizing procedural planning and patient safety in the evolving landscape of mitral valve interventions.

## 1. Introduction

Mitral regurgitation (MR) remains the most common valvular disease affecting 2–3% of the general population and up to 10% of elderly individuals [1]. Regarding moderate–severe MR the global prevalence appears to be 0.67%, increasing markedly with age [2]. It is well established that the relationship between MR severity and one-year mortality is direct, independent and approximately linear [3]. MR presents with two key phenotypes, primary-degenerative MR and secondary-functional MR. The latter one includes both the classical ventricular form and atrial functional MR (AFMR), a recently recognized and pathophysiologically distinct entity occurring in the context of mitral annular dilatation in atrial fibrillation (AF), supported by a growing body of mechanistic and imaging evidence and further described by expert consensus articles [4,5,6].

In the context of MR, stroke remains one of the most devastating complications across its natural history as identified by large-scale epidemiological cohorts and mechanistic studies summarized in Table 1. Beyond the established causal link between AF and cerebrovascular events, the development of distinct atrial cardiomyopathy contributes to the prothrombotic milieu observed in patients with mitral valve disease. Progressive left atrial (LA) enlargement associated with larger regurgitant volume, blood stasis, impaired atrial contractile function and endothelial dysfunction, appear to be culprits for thrombi formation [5,6,7]. The thromboembolic risk in MR follows a dynamic trajectory across the therapeutic continuum.

**Figure 1:**
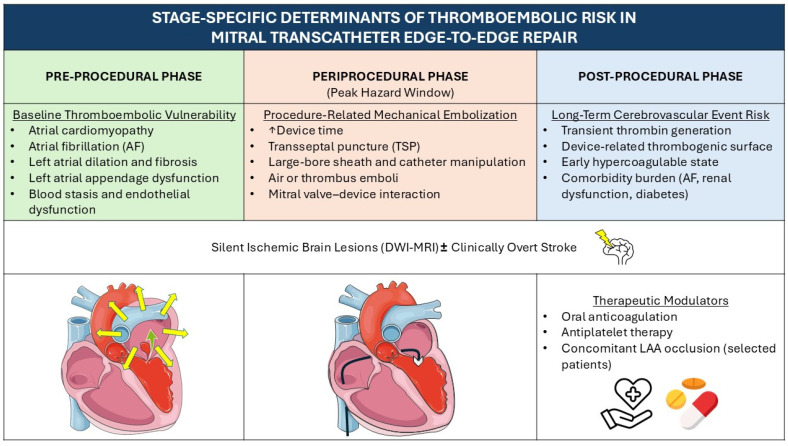
Stage-specific determinants of thromboembolic risk in mitral transcatheter edge-to-edge repair. Abbreviations: AF: atrial fibrillation; DWI-MRI: diffusion-weighted magnetic resonance imaging; LAA: left atrial appendage; TSP: transseptal puncture.

Pre-procedurally, the risk is primarily dictated by the presence of AF and the underlying atrial cardiomyopathy. During the periprocedural phase of mitral transcatheter edge-to-edge repair (M-TEER), mechanical factors such as device manipulation and transseptal puncture (TSP) introduce acute embolic challenges. Ultimately, in the post-procedural period, the clinical focus shifts toward establishing an optimal antithrombotic regimen to prevent device-related thrombosis and ensure long-term safety [12,13,14] (Figure 1).

While individual components of cerebrovascular risk have been described, relative evidence remains fragmented with studies focusing on isolated windows of M-TEER patients’ continuum; thus, a comprehensive synthesis, which maps the longitudinal trajectory of the M-TEER patients, currently lacks in the literature. This review addresses this gap, establishing a unified framework of cerebrovascular risk in M-TEER throughout the entire clinical course. Given the rapid expansion of M-TEER, a structured understanding of stroke mechanisms is needed, to characterize pathophysiological vulnerabilities, navigate potential hazards, and outline future direction for stroke prevention on M-TEER candidates.

## 2. Review Methodology

A dual-database search in PubMed/MEDLINE and Scopus for peer-reviewed articles published between January 1991 and March 2026 was performed and supplemented by backward citation tracking (snowballing) to ensure the inclusion of pivotal mechanistic data, that might have fallen outside the initial keyword-based parameters, while minimizing selection bias. The search timeframe was specifically designed to capture the evolution of the edge-to-edge concept, from the initial interventional descriptions to contemporary transcatheter innovations. The search strategy employed a combination of MeSH terms (Mitral Valve Insufficiency, Transcatheter Mitra Valve Repair, Atrial Remodeling, Thromboembolism OR Cerebral infraction OR Brain Ischemia) and targeted keywords (M-TEER/TMVR, atrial functional MR, silent brain injury, periprocedural risk, microembolic signals, antithrombotic regimen, future directions).

To address the inherent heterogeneity of the literature and ensure methodological transparency, evidence was stratified into three distinct tiers:

Group A (Clinically Actionable Evidence): Randomized controlled trials, multicenter registries, and international guideline documents were prioritized to establish clinical safety endpoints and absolute event rates

Group B (Mechanistic Determinants): Prospective observational cohorts and mechanistic imaging studies provided the foundation for pathophysiology analysis, procedural determinants evaluation and periprocedural embolic risks characterization.

Group C (Hypothesis-Generating Frameworks): Expert consensus documents and viewpoints were utilized selectively to highlight expert perspectives on emerging trends.

Case reports, conference abstracts and posters, non-peer reviewed pre-prints, experimental models, studies lacking neurological/embolic endpoints and duplicate publications were excluded.

## 3. Pre-Procedural Risk: Atrial Fibrillation and Mitral Regurgitation/Atrial Functional Mitral Regurgitation

The burden of AF in patients with MR is high, as it affects 30–50% of those with-moderate to severe disease, showing even higher rates in patients with atrial functional mitral regurgitation (AFMR) [4,9].

The pathophysiologic foundation of thrombogenicity in MR and AF is the sustained volume overload imposed on the left atrium (LA), leading to atrial remodeling. LA dilation leads to increased wall stress, interstitial fibrosis and cellular architecture disruption. Normal atrial function, including reservoir, conduit and booster pump phases, is impaired, leading to blood stasis. These events form the substrate predisposing MR patients to thromboembolic events [10,15,16].

In this context, AF should be regarded as a clinical manifestation of the atrial cardiomyopathy rather than the sole cause of thrombus formation. When AF coexists with MR the remodeling process is further accelerated, posing a bidirectional amplification of thromboembolic risk. In AF, the “booster pump phase” of the cardiac cycle is eliminated, leading to reduced LA emptying velocity, perpetuating LA volume overload [17]. AF begets AF by creating more fibrosis and conduction heterogeneity, while the mechanical desynchrony caused by AF worsens the regurgitant volume and MR severity [17,18,19,20]. Interestingly, older studies suggested that severe MR might even be protective by increasing LA washout and decreasing spontaneous echo smoke [21]. However, more recent meta-analyses have demonstrated that there is no association between MR severity and risk of thromboembolism [22].

AFMR represents a distinct phenotype of MR in which left ventricular function is normal and mitral valve leaflets and subvalvular apparatus are intact. The regurgitation results from mitral annular dilatation and loss of atrial contractility. It is characterized by marked LA enlargement, flattening and dilatation of the mitral annulus and reduced reservoir and conduits strain, being essentially a marker of advanced atrial disease [23,24]. Compared to degenerative MR, AFMR has worse survival and more heart failure hospitalizations, while these patients are less likely to undergo interventions [25]. However, up to date, there are no trials comparing the prevalence of stroke in patients with AFMR vs. VFMR.

These mechanistic insights have clear implications for pre-procedural anticoagulation. Current guidelines recommend oral anticoagulation (OAC) based on CHA_2_DS_2_-VA score, with direct oral anticoagulants preferred over vitamin K antagonists [26,27]. In patients with MR and particularly AFMR, reliance on CHA_2_DS_2_-VA score might underestimate the thromboembolic risk in patients with borderline score and severe LA dysfunction. Despite the absence of substantial prospective evidence, the premise that patients with severe atrial remodeling may benefit from a lower threshold of initiating anticoagulation, particularly in the context of M-TEER, remains clinically relevant and warrants further investigation [28]. Adequate anticoagulation of minimum three weeks or imaging-guided exclusion of LAA thrombus is also recommended, based on current studies and guidelines [26,27,29].

## 4. Procedural Risk: Stroke and M-TEER/TMVR

Despite rigorous pre-procedural risk stratification and antithrombotic optimization, periprocedural stroke represents a critical adverse event of transcatheter mitral valve interventions, with a profound impact on their clinical outcomes [30]. Notably, a significantly lower incidence of clinically overt stroke with M-TEER compared to surgical treatment has been established in the EVEREST-II trial [31]. Building upon these findings, the COAPT and MITRA-FR trials further evaluated the safety of transcatheter repair against optimal medical therapy, reporting low rates of procedural stroke despite their contrasting results regarding long-term clinical benefits [32,33].

The key-conclusion derived from the available pivotal clinical trials [31,32,33] is a consistently low incidence of clinical overt stroke following mitral interventions (Table 2). Τhis finding, however, stands in contrast to the high prevalence of subclinical embolization demonstrated in smaller, mechanistic studies [34,35] utilizing MRI and transcranial Doppler, further highlighting the existing discrepancy between surrogate embolic markers and clinically meaningful neurologic events. The exact incidence of subclinical brain injury during mitral valve interventions is yet not precisely identified as only a few small-scale relevant studies have been demonstrated [36,37]. According to these relevant observational/prospective studies, new ischaemic brain lesions occurred in up to 86% of patients (approaching 100% when systematically evaluated) after M-TEER with the MitraClip system [34,35,38,39]. These studies primarily involved elderly patients (median age: 80 years) with heart failure and moderate to severe MR treated with M-TEER using the MitraClip system. The new ischemic areas, regardless of their magnitude, were verified through the use of pre- and post-procedural diffusion-weighted MRI (DWI-MRI) and continuous transcranial Doppler monitoring during M-TEER. The clinical manifestation of these periprocedural embolic events ranges from asymptomatic silent lesions, detectable only through brain imaging, to clinical overt stroke with measurable neurological deficits [40]. Τhus, while the incidence of subclinical embolization is high, its short-term clinical impact appears to vary and remains a subject of ongoing investigation [37].

Specifically, in Blazek et al.’s study [35], whose aim was to assess the incidence and impact of cerebral embolic events after the M-TEER procedure, the conclusions aimed that although the procedure results in new ischaemic cerebral lesions in the vast majority of patients [23 out of 27 (85%)], these lesions are clinically without significant impact on global cognitive function. Patients in this study (n = 27) underwent MRI examination pre- and post-intervention and their neurological function was evaluated via MoCA test by person blinded to the MRI results. No significant differences were observed in the pre-interventional testing of cognitive function, but a sub-group analysis indicated that patients with >3 new postinterventional embolic lesions (n = 13, 48%) exhibited lower post-interventional MoCA score in comparison to patients with ≤3 embolic lesions. Notably, neither group of patients showed a statistically significant decline in post-interventional MoCA score relative to their baseline score. These findings highlight the ‘hidden fingerprints’ of such invasive procedures [41], where radiographic silent ischemia remains the predominant finding while underscoring the difficulty to establish a definite link between surrogate imaging markers and measurable cognitive decline.

Conversely, in Braemswig et al.’s study [34], whose aim was to identify the procedural step(s) that are associated with an increased risk of cerebral embolisation during M-TEER, conclusions aimed that non-disabling stroke [defined as a mild neurological deterioration with a NIHSS score change < 3] occurred in 9 of 54 patients (17%) after the intervention, which is significantly different from Blazek et al.’s [32] findings. Patients in the Braemswig cohort (n = 54) underwent MRI examination pre- and post-intervention, continuous transcranial Doppler examination during M-TEER to detect microembolic signals (MES) and neurological examination using NIHSS and MoCA score. This disparity in clinical outcomes underscores the ongoing debate regarding whether these complications are truly asymptomatic or if they represent a cumulative neurological burden that warrants further investigation.

Despite the apparent discrepancies in these findings, a critical parameter must be considered when attempting their comparison. Global cognitive screening using the MoCA score, exclusively used in Blazek et al. study [35], did not reveal a statistically significant decline in cognitive function after M-TEER, while higher sensitivity examination by neurologists (NIHSS), added to Braemswig et al.’s [34] study, detected mild deteriorations. Considering that in the former study, patients with >3 new postinterventional embolic lesions presented a declined post-interventional MoCA score in comparison to patients with ≤3 embolic lesion, further neurological assessment could show a mild deficit too. Notably, both studies demonstrated the same incidence of new ischemic lesions (~85–87%).

Thus, this observed divergence in clinical event rates between the cohorts of Blazek et al. and Braemswing et al. presents a complex critical paradox. While these differences phenomenally could be attributed to the varying sensitivity of the neurological assessment tools employed, other confounding variables (including potential differences in baseline patient risk profiles, procedural complexity or periprocedural anticoagulation protocols) may equally contribute to these divergent findings. Consequently, these studies collectively underscore the challenges in characterizing periprocedural neurological injury and underly the necessity for standardized, prospective multicenter validation.

Despite some variability in the reported statistical data, the overarching finding remains consistent: the peak hazard for a cerebrovascular event is concentrated within the periprocedural window, with the risk attenuating substantially thereafter [42]. This risk is distinct from the patient’s baseline embolic potential, originating primarily from factors related to the technical execution and the interaction of the device with the cardiac anatomy. These factors include sheath and catheter dynamics (manipulation, size and stiffness of guides) as well as TSP access technique [43,44]. Moreover, technical complexity and procedural duration, often correlated with the number of clips needed and the patient’s age, remain critical determinants of acute outcomes as stated in large-scale registries [45,46]. In contrast to TAVR, where embolic debris is predominantly calcific, the embolic material encountered during mitral procedures is more heterogeneous, typically consisting of organized thrombus, leaflet tissue, or air bubbles [47,48]. Thus, this distinct pathophysiological profile underpins the current academic debate regarding the clinical efficacy of cerebral embolic protection in the mitral position; as current filtration systems are primarily engineered for calcific sequestration, their capacity to mitigate the risk from non-calcific or gaseous embolic loads remains unproven [48].

The successful and safe delivery of M-TEER systems fundamentally relies on the achievement of LA access via TSP. While a well-established technique, this critical initial step represents a distinct and non-negligible source of periprocedural stroke primarily through mechanical and embolic mechanisms [43,44]. Solely, the mechanical manipulation of the needle, wire and large-diameter sheath complex across the interatrial septum carries a potential of thrombus dislodgement and generation. This risk is amplified by several factors and has been described in certain studies, the findings of which are presented in Table 3. Simultaneously, due to the compromise of the integrity of LA, a significant risk of air entry and subsequent cerebral embolism (micro- or macro-) is presented, which is primarily associated with catheter exchanges. As the TSP stroke danger is well-described, air management protocols and relevant procedural guides aim to minimize the risk via fluid flashing and minimal exchange workflow [44]. Furthermore, the integration of continuous US guidance during the procedure ensures more precise movements aiding in the avoidance of thrombus dislodgement and optimizing the safety [43]. In this context, the choice of anesthesia is pivotal; while conscious sedation has gained ground in other transcatheter interventions, the necessity for high-quality, stable echocardiographic guidance in mitral procedures often dictates the use of general anesthesia to ensure maximum procedural precision and minimize embolic complications [45].

About procedural parameters that are likely to cause periprocedural thromboembolic events, all the relative studies seem to align. Following transcatheter mitral intervention, it has been demonstrated that the procedural duration is a key determinant of embolic risk. Specifically, evidence suggests that the number of Doppler embolic signals differed significantly between the procedural steps with the highest numbers observed during device interaction with the mitral valve (i.e., crossing of the mitral valve, grasping the mitral leaflets and closing the device) which is referred to as device time (*p*-value < 0.0001) [34].

The numbers of clips needed and the degree of mitral insufficiency reduction exhibit a mild–significant trend towards a higher number of lesions [42]. While both factors may appear independent, their association is evident: greater MI severity imposes more clips, which directly increases device time. Though the number of clips is considered an irrelevant independent risk factor, its necessity, alongside the degree of MI reduction, ultimately converges to prolong the device time which seems to be the critical factor associated with ischemic lesions [34,45].

Besides this, the patient’s pre-existing risk factors, particularly AF and CHA2DS-VaSc score, also contribute to periprocedural stroke risk [42,46]. Given that a larger degree of mitral insufficiency is related to more significant rates of AF, the classification of MI pre-procedure seems to be irrelevant to the number of clips and device time parameter of periprocedural stroke risk. Consequently, device time appears to be an independent predictor for the number of post-procedural new lesions and the grade of neurological clinical deterioration respectively [34,35]. Thus, device interaction with the mitral valve is a key target of future improvements of the M-TEER procedure to reduce embolization [50,51].

## 5. Post-Procedural Risk: Lifetime Management of Stroke in Patients with TEER/TMVR

The lifetime management of cerebrovascular risk after transcatheter mitral interventions (M-TEER and TMVR) is one of the main outcomes of long-term clinical trials. Although both procedures demonstrate safety profiles and low stroke rates, recent studies highlight that cerebrovascular risk depends on a complex interaction between comorbidities, periprocedural physiology, early post-procedural hypercoagulability and antithrombotic treatment [42,52]. Analyses have shown a lower incidence of post-procedural stroke in TMVR compared to surgical mitral valve repair and a similar risk when compared with optimal medical therapy alone [42,52]. These findings underscore that catheter-based mitral interventions may reduce perioperative cerebral embolic exposure in contrast with surgical approaches, although without eliminating stroke risk, which remains clinically relevant throughout follow-up.

The COAPT trial [32] provides data about early and late cerebrovascular events (CVEs) after M-TEER. Specifically, across 614 randomized patients, 50 CVEs occurred in 48 individuals (7.8%), with similar incidence between M-TEER plus guideline-directed medical therapy (GDMT) and GDMT alone (12.3% vs. 10.2%; *p* = 0.91), and very low 30-day CVE rates (0.7% vs. 0%; *p* = 0.15). Subgroup analyses suggested that baseline anticoagulation status influenced outcomes: patients receiving OAC at baseline experienced lower rates of cerebrovascular events compared with those not anticoagulated. M-TEER was associated with a marked reduction in cerebrovascular risk only in anticoagulated individuals, whereas the absence of anticoagulation was associated with increased risk. These findings emphasize the vital role of periprocedural and early post-procedural antithrombotic treatment, particularly in patients with AF, renal dysfunction, or diabetes [53,54].

Across contemporary cohorts, the incidence of cerebrovascular events remains low, but the majority of events occur after hospitalization, highlighting the importance of early outpatient risk phase [55,56]. The short-term post-procedural OAC for approximately 30 days led to a reduction in early stroke risk (0.2% vs. median 1.3%; *p* < 0.05) without increasing bleeding complications (4.6% vs. 7.4%; ns), supporting the consideration of anticoagulation after hospital discharge [55]. Meta-analytic evidence further strengthens the case for anticoagulation in select high-risk patients. A pooled analysis of five M-TEER cohorts including approximately 1.900 patients demonstrated that at least four weeks of anticoagulation was associated with a significantly lower stroke rate (RR 0.14; *p* = 0.02) without increasing bleeding risk or mortality [55]. Despite these findings, nearly 40% of patients receiving chronic anticoagulation prior to M-TEER are discharged without it, which is associated with higher mortality (HR 3.84; 95% CI 2.33–6.33) [54]. Furthermore, dual antiplatelet therapy (DAPT) appears equally effective with anticoagulation for stroke prevention in non-AF patients. In patients with an OAC indication, withholding antiplatelet therapy (APT) reduces bleeding by 45% without increasing thrombotic risk [57,58].

Following M-TEER, the coagulation system exhibits a transient activation, with markers such as prothrombin fragment 1 + 2 and thrombin–antithrombin complexes peaking within 24 h and normalizing by one month. In contrast, platelet activation markers, including soluble P-selectin and sCD40L, remain largely unchanged, suggesting minimal platelet-driven thrombogenic post-procedural risk. These results are from a prospective study [59] including 46 patients undergoing M-TEER, indicating that routine prolonged APT may not be necessary after M-TEER, as the prothrombotic risk depends most on thrombin generation rather than platelet activation. Consequently, short-term early anticoagulation strategies warrant consideration because they may mitigate the bleeding risk associated with extended DAPT [26,27], but the clinical efficacy and safety of this strategy remain to be further established.

Left atrial appendage occlusion (LAAO) alongside M-TEER offers procedural success and safety profiles comparable to isolated M-TEER and shows an additional dimension to lifetime cerebrovascular protection. Early studies demonstrate that combining LAAO with M-TEER is feasible and this approach does not introduce incremental periprocedural risk, despite the additional LA intervention. Importantly, both groups report a reduction in long-term OAC use, with OAC use at discharge falling to 9.5% following combined LAAO+M-TEER compared with 86.2% after M-TEER alone, while bleeding risk decreased significantly without compromising ischemic protection [27,59]. Thus, LAAO performed alongside M-TEER may represent a valuable addition to lifetime cerebrovascular protection strategies to high-risk patients after OMT, but data are still limited and mostly observational, so future efforts are needed to determine the safety and efficacy of combined procedures, evaluate technical considerations, and identify the patient phenotype that benefit the most from such an approach [60].

Given the evolving evidence from clinical trials, mechanistic analyses and real-world registries (Table 4), post-procedural cerebrovascular management after M-TEER and TMVR is increasingly conceptualized as a lifetime, individualized process rather than a short-term consideration. Optimal management requires early assessment of stroke and bleeding risk, wise use of temporary anticoagulation and consideration of LAAO in high-risk patients. As the field of transcatheter mitral intervention continues to expand, future randomized trials focusing on antithrombotic strategies will be essential to clarify protocols and practices. However, current evidence supports a personalized, risk-based approach to achieving durable cerebrovascular protection in patients undergoing M-TEER [61].

## 6. Future Directions

Nowadays, transcatheter mitral valve interventions are expanding rapidly, hence ongoing efforts should focus on reducing procedural risk, improving device durability and safety, and optimizing post-procedural management.

Potential improvements to further mitigate the risk of cerebrovascular events peri-procedurally focus on technological advancements. Currents efforts prioritize enhancing the precision and safety profile of TSP through advanced visualization and fluoroless approach in order to overcome the inherent limitations of traditional fluoroscopy-guided access [43,44]. Νewer device generations (Mitraclip G4) are designed to minimize repeated grasping attempts, ensure better sealing and thus reduce device time [34,43,62]. Moreover, procedural safety may improve if future devices reduce reliance on transapical access, become less invasive and are better adapted to different mitral anatomies [63]. Procedural safety is further ensured via enhanced anticoagulation strategy, with strict intra-procedural activated clotting time (ACT) monitoring (with an ACT goal >300 s) which prevents thrombus formation on the delivery system and catheters [49,51]. The integration of intracardiac echocardiography and advanced electroanatomical mapping systems has already enabled fluoroless transeptal puncture [43,44]. Furthermore, the implementation of augmented reality holds the potential to minimize misalignment errors while the adoption of robotic catheter control systems demonstrated initial safety and feasibility [44].

Simultaneously, the emerging paradigm of digital twins—conceptualized as multi-scale, high-fidelity e-replicas of a patient’s specific cardiac anatomy—represents a promising framework with the potential to transform precision intervention through patient-specific in silico simulations and personalized procedural planning. By optimizing clip trajectory and landing zones facilitating in a virtual environment, this technology aims to minimize iterative grasping maneuvers and thus curtail the embolic hazard associated with prolonged device time [64,65]. Nevertheless, whether these technological advancements—including the rapidly evolving field of artificial intelligence, digital twins and robotics—translate into superior clinical outcomes remains a subject of ongoing investigation [65,66], as only limited and small-scale prospective data are available at this moment, necessitating large-scale randomized validation to determine whether these innovations can transition from hypothesis-generating frameworks to clinically actionable tools.

Post-procedural care is also of great importance due to late serious complications after TMVR. It is essential to individualize anticoagulation treatment, optimize medical therapy, and ensure early detection of complications (residual MR, hemolysis, heart failure, arrhythmia) [67,68]. Hence, it is vital to establish standardized post-procedural follow-up protocols (imaging, anticoagulation, medical therapy) based on prospective registries or studies, and to investigate optimal antithrombotic regimens post-TMVR (especially in patients with AF or prior LAA interventions) to minimize the risk of thrombus formation without excessive bleeding. Finally, another matter of importance is better patient selection. It remains critical to refine criteria to determine which patients will benefit most from M-TEER vs. TMVR, balancing the risk of periprocedural complications against the chance of durable MR correction. As noted in recent reviews, and stated in current guidelines [26], patient selection is currently among the major limitations [63]. Thus, developing comprehensive risk stratification models, including clinical, anatomical, imaging, and patient-specific factors, will help guide the choice between repair vs. replacement, or conventional surgery vs. transcatheter approaches.

## 7. Conclusions

The thromboembolic landscape in patients with MR is characterized by a dynamic risk profile that shifts across the different stages of patient care and treatment options. Thromboembolic protection should ideally begin with the early identification of atrial cardiomyopathy—identified as a silent driver of pre-procedural stroke risk—, extend beyond the prevention of periprocedural subclinical ischemic damage in the catheterization laboratory, and result in a post-procedural, personalized long-term antithrombotic treatment. Nevertheless, expanding from the personalized to the digitalized medicine era, tools such as artificial intelligence and digital twins are postulated to play a decisive role in the evaluation of patient risk at different stages of their treatment and assist in selection of the optimal approach for the prevention and management of such events throughout their lifetime.

## 8. Study Limitations

Several limitations of the present review should be acknowledged. First, due to the narrative nature of this synthesis, this work is inherently subject to the authors’ interpretation and evidence weighting, which may introduce a degree of selection bias. Second, the available literature is characterized by marked heterogeneity regarding embolic signals’ detection sensitivity, neurological endpoint definitions and follow-up protocols, thus complicating the direct comparison of stroke incidence across different trials. Furthermore, a significant portion of the evidence is derived from observational registries and relevant analyses, which are susceptible to inherent confounders. Finally, given the rapidly evolving technological landscape of transcatheter interventions and AI integration, the insights provided on this review reflect the current evidentiary status, which remains subject to future validation by ongoing large-scale randomized controlled trials.

## Figures and Tables

**Table 1:** Overview of landmark studies establishing the association between mitral regurgitation and thromboembolic risk.

Study/Year	Key Finding	Mechanistic Insight
Avierinos et al. (2002) [8]	Organic mitral regurgitation (DMR) is associated with a sustained stroke risk (~2% per annum).	Left atrial (LA) diameter >50 mm serves as an independent predictor of embolic events, even in sinus rhythm.
Benjamin et al. (Framingham Heart Study) (1994) [9]	Significant correlation between MR severity and the development of atrial fibrillation (AF).	Chronic volume overload induces structural and electrical LA remodeling, creating the substrate for AF.
SPAF Investigators (1992) [10]	MR functions as a thromboembolic risk modifier in patients with non-valvular AF.	Chronic MR promotes LA stasis and spontaneous echo contrast (SEC), counteracting the “washout effect” of the regurgitant jet.
Grigioni et al. (2001) [11]	Severe ischemic MR is independently linked to a higher rate of thromboembolic complications.	Left ventricular dysfunction exacerbates stagnant blood flow within the atrial and ventricular cavities.

Abbreviations: AF: atrial fibrillation, LA: left atrial, MR: mitral regurgitation.

**Table 2:** Summary of studies evaluating the occurrence of embolic events (clinical and silent) and their relationship with the overall outcomes of the M-TEER intervention or surgical management of mitral regurgitation.

Study (Year)	Design/Population (N)	Endpoint Type/ Assessment	Key Finding	Novel Insights (Calibrated)	Major Limitations
Large RCTs		Hard clinical endpoints			
Stone et al. (COAPT Trial) (2018) [32]	RCT (MitraClip vs. GDMT) (N = 614)	Clinical follow-up (Adverse Events)	Low incidence of clinically overt stroke (0.2% to 1.2% at 30 days).	Established the clinical benefit and the low clinical stroke risk of M-TEER in secondary MR	Highly selected population, not designed to detect subclinical embolic events.
Feldman et al. (EVEREST-II Trial) (2011) [31]	RCT (MitraClip vs. Surgery) (N = 570)	Clinical follow-up (Adverse Events)	Low stroke incidence, comparable or superior to surgical safety at 30 days.	Established M-TEER as a viable alternative to surgery from a safety perspective	Early generation device, limited sensitivity in capturing subclinical embolic load.
Obadia JF et al. (MITRA-FR Τrial) (2018) [33]	RCT (Mitraclip vs. OMT) (N = 304)	Clinical follow-up (Adverse Events)	No significant difference in death or HF hospitalization (54.6% vs. 51.3% for OMT alone, *p* = 0.53).	Suggested that clinical benefits may be limited in patients with massive LV remodeling.	Primary focus on long-term clinical outcomes (HF/Death); absence of periprocedural neurological assessment.
Mechanistic and observational		Surrogate/radiographic endpoints			
Braemswig et al. (2022) [34]	Prospective/Observational (N = 54)	Surrogate (TCD for MES/DWI-MRI)	MES in 100% of patients. New DWI lesions were detected in 21/24 patients. MV–device interaction yielded the highest number of MES.	Identified MV–device interaction as the most vulnerable procedural step for cerebral embolization.	Small sample size; clinical significance of silent lesions remains unproven.
Blazek et al. (2015) [35]	Prospective/Observational (N = 27)	Surrogate (DWI-MRI)	New ischaemic lesions (silent ischemia) occurred in up to 86% of patients.	Highlighted the high incidence of silent brain ischemia despite low overt strokes.	Single-center; very small sample size; no long-term neurocognitive follow-up.
Barth et al. (2017) [38]	Prospective, Observational (N = 26)	Surrogate (DWI-MRI)	New lesions were found in 77% of patients, confirming the high rate of silent embolization.	Confirmed the high frequency of silent ischemia and stated the “clinical-radiographic paradox” in M-TEER.	Observational nature; lack of a control group.
Frerker et al. (2016) [39]	Prospective, Observational (N = 36)	Surrogate (Debris vis CEP)	Embolic debris was captured in the CEP filter in 100% of examined patients.	Demonstrated the presence of embolic material; suggests potential utility of CEP.	Feasibility study; cannot prove clinical efficacy in stroke reduction.

Abbreviations: CEP: cerebral embolic protection, DWI-MRI: diffusion-weighted magnetic resonance imaging, LV: left ventricle/left ventricular, MES: microembolic signals, M-TEER: mitral transcatheter edge-to-edge repair, MV: mitral valve, OMT: optimal medical treatment, RCT: randomized controlled trial, TCD: continuous transcranial Doppler.

**Table 3:** References related to the mechanisms of periprocedural stroke incidence.

Study	Documented Mechanism/Relationship with Stroke
Barros da Silva et al. (2020) [42] Meta-analysis/1881 patients	Thrombosis/Hemodynamic Disturbance: Documents stroke risk due to altered flow dynamics (blood stasis) and the new double-orifice created post-MitraClip.
Denysiuk et al. (2024) [43] State-of-the-art Review	TSP Technique and Embolism: Documents the technical and anatomical complexity of TSP as a source of ischemic events/TIA and stresses the need for uninterrupted anticoagulation.
Nusca et al. (2018) [49] Systematic Review	Mechanical Embolism: States that early embolic events are linked to large-bore catheter manipulation and the dislodgement of particulate debris/air during the procedure.
Guicciardi et al. (2025) [44] Detailed Review	TSP Technique: Detailed analysis of TSP complications, including the role of TEE guidance in avoiding wrong punctures and subsequent air/thrombus emboli.
ACC/AHA/ESC Guidelines	Periprocedural Anticoagulation: Establishes the standard unfractionated heparin protocol (target ACT) for minimizing thrombus formation on the catheter/sheath surface during the structural procedure.

**Table 4:** Summary of studies on cerebrovascular events after TEER and TMVR.

Study	Type of Study	Population/Procedure	Key Findings	Novel Insights (Calibrated)
Da Silva et al. (2020) [42]	Observational/Comparative	TMVR vs. Surgical mitral repair	Lower post-procedural stroke in TMVR vs. surgery; similar to optimal medical therapy	Highlights importance of comorbidities, periprocedural physiology, early hypercoagulability
Wang et al. (2023) [52]	Observational	TMVR/M-TEER	Catheter-based interventions are associated with a potential reduction in perioperative cerebral embolic exposure; long-term stroke risk remains a significant clinical concern	Anticoagulation remains a pivotal component of long-term therapy
Vincent et al. (COAPT) (2023) [12]	Randomized controlled trial	M-TEER + GDMT vs. GDMT alone, 614 patients	7.8% CVEs overall; very low 30-day CVE (0.7% vs. 0%)	M-TEER reduces CVE mainly in anticoagulated patients; emphasizes periprocedural antithrombotic treatment
Geis et al. (2020) [51]	Observational	M-TEER patients with AF, renal dysfunction, diabetes	Patients not receiving anticoagulation seem to be predisposed to an increased risk of CVEs	Anticoagulation is strongly advocated in high-risk groups
Hohmann et al. (2022) [54]	Observational	M-TEER	Reinforces increased CVE risk without anticoagulation	Early post-procedural anticoagulation warrants careful consideration
Zhang et al. (2023) [55]	Prospective cohort	M-TEER/TMVR, contemporary patients	Most CVEs occur post-hospitalization; early anticoagulation (~30 days) reduces early stroke (0.2% vs. 1.3%)	Short-term anticoagulation demonstrated a favorable safety profile, with no significant increase in major bleeding events
Frazzetto et al. (2023) [56]	Observational	M-TEER/TMVR	Majority of CVEs occur after discharge	Emphasizes outpatient risk management
Dimitriadis et al. (2024) [60]	Retrospective cohort	M-TEER, chronic anticoagulation prior	40% discharged without anticoagulation; higher mortality (HR 3.84)	Continuation of anticoagulation post-discharge is recognized as a key component in long-term risk mitigation
Claeys et al. (2024) [57]	Observational	M-TEER, non-AF patients	DAPT equally effective as anticoagulation for stroke prevention	Withholding antiplatelets in OAC patients has been associated with reduction in bleeding without raising thrombotic risk
Al-Abcha et al. (2025) [58]	Observational	M-TEER, OAC patients	Confirms Claeys et al.	Supports reduced bleeding without increasing thrombotic risk
Hadjadj et al. (2025) [59]	Prospective mechanistic study	M-TEER, 46 patients	Transient thrombin generation; platelet activation unchanged	Short-term anticoagulation may be adequate, whereas the incremental benefit of prolonged APT remains to be established
Ahmad et al. (2025) [30]	Observational/Early feasibility	M-TEER + LAAO	Procedural success similar to Μ-TEER alone; long-term OAC reduced	LAAO reduces bleeding while maintaining ischemic protection
Hadjadj et al. (2025) [59]	Observational	M-TEER + LAAO	Confirms Ahmad et al.	Combined procedure safe and effective in high-risk patients
Dimitriadis et al. (2025) [61]	Review/Expert consensus	M-TEER/TMVR	Supports lifetime, individualized CVE management	Early risk assessment, temporary anticoagulation, consider LAAO in selected patients

Abbreviations: M-TEER: mitral transcatheter edge-to-edge repair, TMVR: transcatheter mitral valve replacement, OAC: oral anticoagulation, CVE: cerebrovascular event, APT: antiplatelet therapy, LAAO: left atrial appendage occlusion, GDMT: guideline-directed medical therapy, DAPT: dual antiplatelet therapy.

## Data Availability

No new data were created or analyzed in this study.

## Abbreviations

The following abbreviations are used in this manuscript:

ACT	Activated Clotting Time
AF	Atrial Fibrillation
AFMR	Atrial Functional Mitral Regurgitation
APT	Antiplatelet Therapy
AR	Augmented Reality
CEP	Cerebral Embolic Protection
CVE	Cerebrovascular Event
DAPT	Dual Antiplatelet Therapy
DWI-MRI	Diffusion-Weighted Magnetic Resonance Imaging
EAM	Electroanatomical Mapping
GDMT	Guideline-Directed Medical Therapy
ICE	Intracardiac Echocardiography
LA	Left Atrium/Left Atrial
LAA	Left Atrial Appendage
LAAO	Left Atrial Appendage Occlusion
MES	Microembolic Signals
MR	Mitral Regurgitation
M-TEER	Mitral Transcatheter Edge-to-Edge Repair
NIHSS	National Institutes of Health Stroke Scale
OAC	Oral Anticoagulation
TAVR/TAVI	Transcatheter Aortic Valve Replacement/Implantation
TCD	Transcranial Doppler
TMVR	Transcatheter Mitral Valve Replacement
TSP	Transseptal Puncture
